# Benefits of antioxidant supplements for knee osteoarthritis: rationale and reality

**DOI:** 10.1186/s12937-015-0115-z

**Published:** 2016-01-05

**Authors:** Ashok Kumar Grover, Sue E. Samson

**Affiliations:** Department of Medicine, HSC 4N41, McMaster University, 1280 Main Street W., Hamilton, ON L8S 4K1 Canada

**Keywords:** Arthritis, Turmeric, Curcumin, Piperine, Avocado, *Boswellia*, Superoxide, Peroxide, Peroxynitrite

## Abstract

Arthritis causes disability due to pain and inflammation in joints. There are many forms of arthritis, one of which is osteoarthritis whose prevalence increases with age. It occurs in various joints including hip, knee and hand with knee osteoarthritis being more prevalent. There is no cure for it. The management strategies include exercise, glucosamine plus chondroitin sulfate and NSAIDs. In vitro and animal studies provide a rationale for the use of antioxidant supplements for its management. This review assesses the reality of the benefits of antioxidant supplements in the management of knee osteoarthritis. Several difficulties were encountered in examining this issue: poorly conducted studies, a lack of uniformity in disease definition and diagnosis, and muddling of conclusions from attempts to isolate the efficacious molecules. The antioxidant supplements with most evidence for benefit for pain relief and function in knee osteoarthritis were based on curcumin and avocado-soya bean unsaponifiables. *Boswellia* and some herbs used in Ayurvedic and Chinese medicine may also be useful. The benefits of cuisines with the appropriate antioxidants should be assessed because they may be more economical and easier to incorporate into the lifestyle.

## Current knowledge

Arthritis may have originated before man itself since it also afflicts other primates [1]. The disease causes disability due to pain and inflammation in joints. There are many different types of arthritis of which rheumatoid arthritis and osteoarthritis (OA) are the most common. Rheumatoid arthritis is an autoimmune disease that affects mainly small joints such as those in the fingers of the hand. OA affects large joints such as hips and knees and also those in the hands. OA is a leading cause of disability with an unknown cause or cure. The global age standardised prevalence of OA in the knee and hip has recently been reported to be 3.8 and 0.85 %, respectively [2]. Worldwide estimates indicate that 9.6 % of men and 18 % of women ≥60 years have symptomatic OA (http://www.who.int/chp/topics/rheumatic/en/). Other risk factors of knee OA include trauma such as torn meniscus, occupation, exercise, gender (more common in females), ethnicity, genetics, obesity, diet and bone density [2]. Since OA normally progresses with age, its economic burden may increase with the aging human population in the coming decades. This review will focus on knee OA which is more common. The knee joints are the largest and being synovial joints, they provide a very high degree of mobility. A knee joint provides two articulations - one between tibia and femur and the other between patella and femur [3, 4]. The joints allow for flexion, extension and a limited degree of rotation. It contains a bone-cartilage interface and a synovial body. The synovial body contains the fluid whose composition and viscosity are key to the knee operation. The bone-cartilage interface is a complex functional unit and biocomposite at the centre of joint function in which the individual components interact cooperatively and synergistically. Due to this intimate contact between bone and cartilage, any changes in either tissue will influence the other component. Its role in knee OA is discussed in the next section.

### Pathophysiology of knee OA

The suggested causes for the development of OA include genetic predisposition, aging, obesity, trauma, and other systemic diseases [5]. Irrespective of the etiology, a number of processes that occur in the initial stages may involve cellular and ultrastructural changes which gradually accompany the phenotypic image of OA. During OA there is a loss of cartilage, the subchondral bone becomes thicker, the subchondral trabecular bone mass decreases and new osteophytes are formed [3]. These changes may lead to the development of bone cysts and lesions in the bone marrow. Subsequently, the cartilage layer may be calficified and cracks may occur in it. The chondrocytes, which are normally quiescent, may also actively proliferate and form clusters. An early-stage increase in the remodelling and bone loss followed by a slow remodelling and subchondral densification are hallmarks of OA pathogenesis [6].

It is commonly stated that the articular cartilage protects the bone to prevent any damage during motion, however, the articular cartilage, subchondral plate and trabecular bone are a biologically and functionally inseparable osteochondral unit which absorbs and distributes loads across the joint [7, 8]. The osteochondral plate consists of the following: a thin layer of cortical bone (also called the subchondral plate), a calcified layer of cartilage, adjacent articular cartilage and the tidemark. The subchondral plate is a highly vascular cortical bone. The entire osteochondral plate should be viewed as an exchange area between bone and cartilage through which the bone supplies the cartilage with nutrients and oxygen. Due to this intimate contact between bone and cartilage, any changes in either tissue will influence the other component. The distance between the two bones is important because a decrease in this distance may increase friction during motion. The muscles and the joint are vascularized and innervated. Thus, changes in the elements in the supplied blood may also influence the properties of the joint. This also means that signals from the nerves may lead to a neuropathic pain in the joint [3].

During OA there is a loss of cartilage, the subchondral bone becomes thicker, the subchondral trabecular bone mass decreases and new osteophytes are formed [3]. It has been shown that the subchondral bone sclerosis is characterized by a trabecular thickening and a decrease in calcium binding to the collagen fibers. This abnormal mineralization is due to the overproduction of the homotrimeric alpha1 form of type I collagen by osteoblasts. This (α1)3 type I collagen has a lower affinity for calcium than the (α1)2 α2 type I collagen [7, 8]. This is consistent with a recent study using Raman spectroscopy which concluded that the chemical compositions and collagen quality were different between subchondral bone in the advanced OA and the non-OA distal femur [9]. There may also be changes in the parathyroid hormone induced cAMP levels, and vitamin D induced production of alkaline phosphatase and osteoclacin [7]. OA osteoblasts produced more insulin-like growth factor-1 and urokinase than normal cells. Other changes in the subchondral osteoblasts in OA may be altered production of interleukin (IL)-6, IL-8, metalloproteases and transforming growth factor (TGF)-β1. Increased Wnt signaling in the subchondral bone can also contribute to OA development [7]. It is reminded that there is a close cross-talk between the various tissues in the osteochondral unit and hence changes in the subchondral bone may affect the cartilage and the reverse may also occur. The precise locale of the changes in OA within this unit remains to be established. These changes may lead to the development of bone cysts and lesions in the bone marrow. Subsequently, the cartilage layer may be calficified and cracks may occur in it. The chondrocytes, which are normally quiescent, may also actively proliferate and form clusters. An early-stage increase in the remodelling and bone loss followed by a slow remodelling and subchondral densification are hallmarks of OA pathogenesis [6].

OA affects the entire joint: the cartilage is damaged, the underlying subchondral bone structure is remodelled, and a chronic inflammation of the synovium develops [4]. There is sufficient evidence that during OA the cross-talk between different tissues in the joint becomes more pronounced [3, 7, 8].

A current thinking is that OA is a chronic inflammation disease that occurs with gradual changes in the immune system. This hypothesis has been presented in detail in a recent review [5]. The progression of OA involves changes in the production and functioning of various cytokines. The cytokines involved may be inflammatory interleukins (IL-1*β*, IL-6, IL-15, IL-17, and IL-18) and tumour necrosis factor-alpha (TNF-*α*) or anti-inflammatory interleukins (IL-4, IL-10,and IL-13). The increase in IL-1 causes damage to the articular cartilage [5]. The effect of TNF-*α* is similar to and synergistic with the actions of IL-1*β*. The net result is a blockage of the synthesis of proteoglycan components, the proteins which bind proteoglycans, and type II collagen in chondrocytes [10, 11]. Activated chondrocytes also produce the matrix metalloproteases MMP-1, MMP-3,MMP-13 [5]. The effect of IL-6 on the cartilage is similar to and in synergy with that of the other inflammatory cytokines and leads to a decrease in the synthesis of type II collagen and an increase in the matrix metalloprotease activity [5, 12]. Serum concentrations of IL-5 have been associated with the severity of pain in OA [13]. However, the concentrations in the synovial fluid are higher in early knee OA patients when compared to end-stage OA [14]. The level of IL-17 increases in the serum and in the synovial fluid. This level in the latter correlates positively with the radiographic image of lesions in OA [15]. IL-18 affects chondrocytes and synovial cells by increasing the levels of several inflammatory compounds [5]. The anti-inflammatory cytokines act primarily by decreasing the levels of inflammatory cytokines, especially IL-1*β* and TNF-α [5]. Thus, there is sufficient evidence to support the immune hypothesis in OA.

### Diagnosis of knee OA

Assessment of efficacy of a therapy requires an understanding of diagnosis of a disease such as OA which is first reported by the patient as pain and inability to perform certain routine physical tasks. The clinician diagnoses it as OA by ruling out other causes such as rheumatoid, psoriatic and septic arthritis, injury and other causes.

The diagnosis of the severity of OA is subjective: based on a quality of life questionnaire, physical examination and radiography (for a summary see [4]). The most commonly used quality of life measure is Western Ontario McMaster Index (WOMAC) although other similar measures such as visual analog scale (VAS) for pain and Lequesne index are also used [16, 17]. An example of the WOMAC questionnaire for the knee is at http://www.orthopaedicscore.com/scorepages/knee_injury_osteopaedic_outcome_score_womac.html. The WOMAC questionnaire contains several parts and contains questions related to severity and frequency of symptoms such as swelling of the joint, grinding and clicking noises, knee catching or hanging up, and the ability to straighten or bend knees, pain in the knees in different positions, knee functions and ability to perform daily functions. Based on the sum of all the scores, the overall WOMAC score is determined. Higher scores indicate greater severity.

The physical exam focuses on the range of motion (both passive and active), muscle strength, ligament stability and tenderness of the affected joints [4]. Any swelling due to inflammation is also examined. Examination by palpation of the OA knee may show the presence of a crepitus (a crackling or crunching sensation on range of motion of the joint).

Diagnosis of OA by imaging techniques has recently been summarised [18]. Traditionally it has been diagnosed with X-ray radiographs from different angles to look for joint space width (JSW) and asymmetry, and for the presence of osteophytes. Grading schemes to describe the severity of the disease based on radiography have been developed but these are questioned by others since this method does not yield much information on changes in soft tissue and cartilage [19]. Therefore, the more recent methods such as magnetic resonance imaging (MRI), ultrasound, and optical coherence tomography, have enhanced the OA diagnosis [18]. However, most clinical trials have not used these methods due to their high cost, low availability and the unavailability of literature establishing their association with other criteria of osteoarthritis. In most trials the assessment of pain and function has been used.

### Current therapies for management of knee OA

There are a large number of treatments of varying efficacy and faith for OA. The non-pharmacological treatments include education, exercise, physiotherapy, weight loss, physical aids (supports, braces and walkers) and surgical joint replacements [16, 20–24]. Massage with and without pharmacological agents may also be beneficial. Vitamins and herbs have also been used. A number of recommendations on the treatments for OA made by the American Academy of Orthopaedic Surgeons (AAOS) are available at the website http://www.aaos.org/Research/guidelines/GuidelineOAKnee.asp.

Physical aids may enable the mobility of the patients and allow them to carry out more physical activity. Thus, even then the aim remains an increased physical activity through some form of exercise. Weight loss is the common mantra of the health care professionals for the management of OA, it is moderately recommended by AAOS but its validity may be questionable [25]. A variety of exercise programs of varying effectiveness are available for knee arthritis. It appears that for OA patients, exercises involving supervised slow movements or isometric exercises may be efficacious and also have a lower possibility of damage to the joint than other exercises [16, 20–23, 26]. Therefore, aquatic exercises, yoga and tai chi should be preferred. This is one of the strongest recommendations from the AAOS. Running on treadmills should be avoided. For less severe OA some exercises with slower movements and greater resistance may be added. Since the type, intensity and dose of exercise may benefit each patient differently, physiotherapy is often used to determine the type and extent of the exercises [23]. It is claimed that primary care physiotherapists and pharmacists may improve short term outcomes for older adults with knee pain and reduce the use of NSAIDs [26]. A long term randomised trial which will monitor the effectiveness of community therapy and enhanced pharmacy review for people over the age of 55 has been initiated [20]. It may better resolve these claims.

Several pharmacological agents have been used for management of OA. Temporary pain relief and hence improvement in function may be obtained with analgesics but this is not specific to OA. NSAIDs are used orally and topically because they have some anti-inflammatory and analgesic effects. They are also strongly recommended by AAOS. However, they may have severe adverse effects upon prolonged use. These issues have been discussed in recent reviews and will not be retraced here [27, 28]. There are several commercial preparations such Instaflex, Sierrasil, hyaluronic acid and Aquamin of limited proven usefulness [29–31]. However, the combination of glucosamine and chondroitin sulfate is the most promising. This treatment may be efficacious for pain relief, functional improvement and also result in less joint space narrowing [32–36].

Herbs have been used for such treatment since ancient times in Indian medicine (Ayurvedic) and Chinese medicine [37, 38]. The use of some herbs is also mentioned in the Bible (http://www.threemagi.com/frankincense.html). Most of these herbs have antioxidant properties: they contain compounds or chemicals that can modulate oxidative metabolism which is altered during OA. Many in vitro studies are available in this area. However, the human body is more complex than the cells cultured in defined growth media. Oxidative stress may play a role in several diseases but the benefits of different antioxidant supplements may be unique to each one. We recently reviewed the literature on the benefits of antioxidants in vision health and in obesity-diabetes II [39, 40]. In vision health, the antioxidant supplements containing vitamin C, vitamin E, lutein, zeaxanthin, zinc and copper have a reasonable probability of retarding age-related macular degeneration but the benefits in other eye diseases are questionable. In obesity and diabetes 2, there are marginal benefits of supplementation with zinc, lipoic acid, carnitine, cinnamon, green tea, and possibly vitamin C plus E. Some of the antioxidants are beneficial for obesity and others are better for glucose level regulation.

## Antioxidant supplements in knee OA

### Reactive oxygen species in OA

A free radical is a molecule with an unpaired electron in its outermost orbit [41, 42]. In biological systems, a free radical that involves oxygen is termed a reactive oxygen species (ROS) but the term ROS is used loosely for oxidants such as peroxides. Normal physiological processes result in the generation of ROS such as peroxide, superoxide, hydroxyl radical and peroxynitrite [41, 42]. Thus, ROS occur normally in the body at very low concentrations (nanomolar to micromolar). They are a necessary evil since our body needs them for survival but, when in excess, they may have deleterious effects. Our body gets rid of the excess ROS using natural antioxidants such as vitamin C (ascorbate), vitamin E, glutathione and various enzymes [41–43]. The term oxidative stress is used as a measure of the overall ROS status. It is the ratio of the amount of peroxide present to that of the antioxidant capacity of the cell. High levels of oxidative stress may damage the cells by oxidising lipids and by altering DNA and protein structure.

**Fig. 1:**
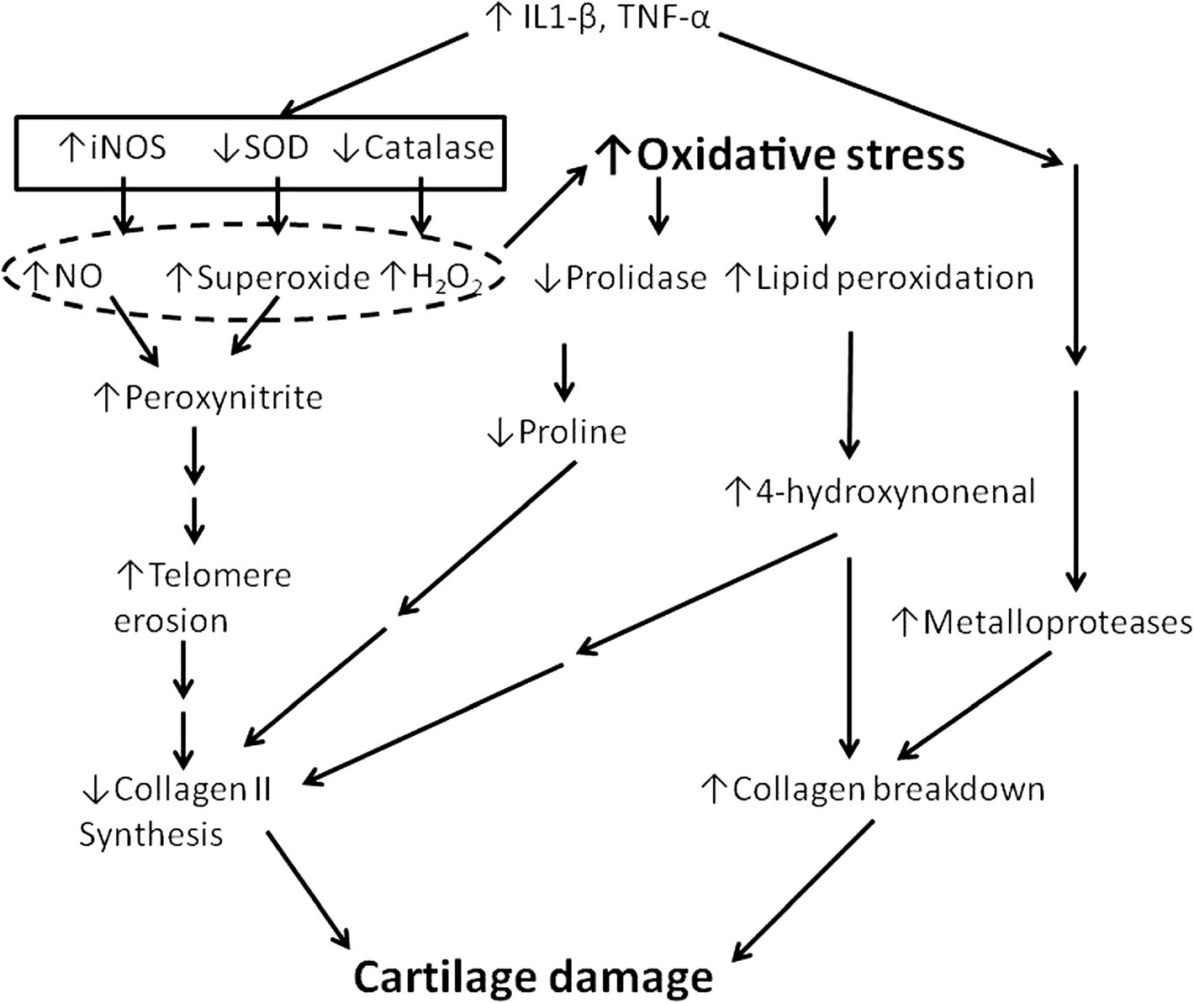
Role of oxidative stress in cartilage damage during OA. OA is hypothesised as a chronic inflammation disease that occurs with gradual changes in the immune system (see Pathophysiology of knee OA). IL-1*β* and TNF-α and other inflammatory factors increase in OA. This pathway leads to induction of NO synthase, production of larger amounts of NO and a deficiency in SOD and catalase (see Role of ROS in OA). The deficiency in SOD leads to higher levels of superoxide which combines with NO to produce peroxynitrite which can cause telomere erosion by targeting guanine repeats in their DNA telomeres. The net result is a decrease in the synthesis of collagen II. The decrease in catalase results in accumulation of peroxide to increase lipid peroxidation which produces 4-hydroxynonenal. The 4-hydroxynonenal increases factors which breakdown collagen II and also inhibits the expression of collagen II. The net result is the cartilage damage that occurs in OA. Note that the scheme shown here is only a summary

The concentrations of different ROS, reduced and oxidised thiols, oxidative stress index and the related enzymes have been monitored in OA. In one study, the serum thiol levels and catalase activity were lower in advanced stage OA patients than in controls and the oxidative stress index was also higher (*P* <0.001, for all) [44] (Fig. 1). In this work the levels of prolidase (a cytosolic exopeptidase which cleaves imidodipeptides and imidotripeptides with C-terminal proline or hydroxyproline) activity correlated negatively with the oxidative stress index. Another study examined the synovial fluid from OA patients undergoing total knee replacement surgery [45]. In these patients, the lipid peroxidation and antioxidants were similar to those with the injured knee joint patients but Vitamin E deficiency was associated with OA [45]. However, a similar study found that the extracellular superoxide dismutase (SOD) levels were lower in the advanced stage OA patients than with the knee injury controls [46]. There was also decline in the concentrations of reduced glutathione and ascorbate [46].

#### The above paradigm of oxidative stress is too simplistic

The kinetic constraints indicate that in vivo scavenging of ROS is ineffective as an antioxidant defense [43, 47]. The concept does not consider that the damage may be unique to each ROS in different cell types. Also, often individual ROS species may act as signals. Therefore, a better concept of oxidative stress is that of a disruption of redox signalling and control [48]. In discussing knee OA, this would be the effects on synovium, cartilage and the joints. These effects relate directly to the OA pathophysiology. For example, IL-1*β* is one of the most active cytokines during the development of OA and it stimulates the production of ROS such as peroxides and hydroxylated radicals and the production of NO and a deficiency in SOD. The deficiency in SOD leads to higher levels of superoxide. NO and superoxide react to form peroxynitrite [46] (Fig. 1). Peroxynitrite can cause telomere erosion by targeting guanine repeats in the DNA. The net result is a decrease in the synthesis of collagen II which is required for the maintenance of the cartilage. Another potential pathway by which ROS can damage the joint is through lipid peroxidation which produces 4-hydroxynonenal. Higher levels of 4-hydroxynonenal are present in the synovial cells of OA compared to those of the healthy subjects [49]. In cartilage explants, 4-hydroxynonenal induced cleavage of collagen II [49]. In chondrocytes isolated from OA patients, 4-hydroxynonenal inhibited the expression of collagen II and increased the levels of factors which can cause its degradation [49]. Thus, the production of 4-hydroxynonenal by ROS could play a major role in OA (Fig. 1). It is reminded that the joint is a system in which the cartilage, the bone, the ligaments and the synovium form a capsule and there is sufficient cross-talk between all the tissues. A diffusion of ROS and lipid peroxidation products may occur between them. Thus, the damage to one element of the joint may influence others through fluid diffusion and by paracrine factors [50].

### Antioxidant supplements and OA

The role of ROS in the pathophysiology of knee OA provides for the rationale that suppressing the ROS levels with the appropriate antioxidant supplements may retard the progress of the disease. What remains to be discussed is the reality of the observations on the effects of such supplements on prevention and/or management of OA.

The effects of food intake and various vitamins and related compounds on OA has been examined and reviewed [51–59]. One concludes that nutritional habits involving fruits, fruit juices and vitamin supplements may be beneficial in the long run but they may not help once OA has already been initiated. Several antioxidant supplements derived from turmeric, avocado, *Boswellia* and other herbs will be discussed.

### Turmeric

Turmeric is used extensively as a spice and has been used for over 4000 years as an Ayurvedic medicine. It is the rhizome of *Curcuma longa* (some studies use *Curcuma domestica*) and contains over 20 different active compounds [60]. Curcumin, a compound with antioxidant properties, was isolated from turmeric about 200 years ago [61]. The properties of curcumin and its potential role in the therapy of several chronic diseases including arthritis, cancer and neuronal disorders have been explored. The rationale for its use in OA comes from several in vitro studies. Curcumin inhibited the matrix degradation of articular explants and chondrocytes [51]. It decreased the production of MMP-3, −9 and −13 via c-Jun-N-terminal kinases, nuclear factor kappa-beta (NFκβ), and the JAK/STAT pathways. It also restored type II collagen and glycosaminoglycan synthesis. A random double blind study on knee OA patients compared the ability of curcuminoids and the NSAID diclofenac to inhibit cyclo-oxygenase 2 [62]. Both the groups significantly reduced (*p* <0.001) cyclo-oxygenase 2 secretions by similar efficacies.

A PubMed search in August 2015 with the words “tumeric/curcumin” and “osteoarthritis” and “knee” showed 21 entries of which 11 were clinical trials involving pain and function with different diagnostic measurements, durations, controls, blindness and conflicts of interests (Table 1). All the trials showed benefits of curcumin except for one in which significance levels were not attained due to a large variance. Some studies have also compared their effects with those of NSAIDs. One randomised double blind study compared the effects of ibuprofen (2 × 400 mg/day) with those of curcumin (4 × 500 mg/day) in patients who were over 50 years of age, had severe knee pain and their radiography showed the presence of osteophytes [63]. Both the groups showed improvements in all assessments but the curcumin group was statistically better in patient satisfaction, timed walk or stair climbing and pain during walking or stair climbing. A similar study with a larger number of patients compared the effects of *C. domestica* extracts (1500 mg/day, *n* = 171) with ibuprofen (1200 mg/day *n* = 160) for 4 weeks [64]. After 4 weeks, all measures of WOMAC and the 6 min walk test improved significantly (*p* <0.001) in both groups with no differences between the groups. The safety profile was found to be somewhat better in the curcumin group. Another study compared the effects of the NSAID diclofenac (75 mg) combined with placebo (*n* = 36) or with *Curcuma longa* extracts (1000 mg) (*n* = 37) on pain and function. The combination was no more effective than diclofenac alone indicating that the effects of the two treatments are not additive (Table 1) [65].

**Table 1:** Knee OA antioxidant supplements based on turmeric, avocado and *Boswellia*

Reference	Study type	Parameters measured	results	Comments
*Turmeric (curcumin)*				
Kuptniratsaikul et al. 2014 [63, 64]	RDB^a^: compared curcumin (*n* = 171) with ibuprofen (*n* = 160)	Thai modified WOMAC, 6 min walk and patient satisfaction	All improved in both groups (*p* <.001) with no intergroup differences	Trial only 4 weeks
				Safety profile better for curcumin
Belcaro et al. 2010 [71]	Open: best available treatment + Meriva versus best available treatment (*n* = 100)	Treadmill walking test, WOMAC and Karnofsky, and oxidative stress levels, inflammatory markers.	8 months - all measures improved with Meriva (*p* <0.05)	Not a blind study
Belcaro et al. in 2014 [66]	Open: Meriva and glucosamine (*n* = 63) vs chondroitin and glucosamine (*n* = 61)	Treadmill walking test, WOMAC and Karnofsky scales	4 month - similar improvements in both groups. Use of NSAIDS decreased in both groups	Not a blind study
Panahi et al. in 2014 [66, 68]	RDBP: curcumin + bioperine (*n* = 21) vs placebo (n =19)	WOMAC, VAS, Lequesne’s pain and stiffness score	6 weeks - improvement in WOMAC, VAS, Lequesne’s pain scores (*p* <.0010) but not in stiffness score	Mild gastrointestinal symptoms reported in both groups
Pinsornsak 2012 [65]	DB: (Curcumin 1000 mg + diclofenac 75 mg)/day (*n* = 44) vs (diclofenac 75 mg + placebo) (*n* = 44)	VAS AND Knee Injury and Osteoarthritis Outcome Score	No difference between groups for pain and function.	Small group size, submaximal doses The effects of two treatments is not additive
Henrotin, Y et al. 2014 [67]	Open: Flexofytol (curcumin with polysorbate) (*n* = 100) in real life situations	Serum Coll-2-1, Coll-2-INO_2_, Fib3-1, Fib3-2, CRP, MPO, CTX-II. VAS pain	6 months - Coll-2-1 decreased. No change in pain	Not a blind study, no control
Appelboom et al. 2014 [72]	Open: Flexofytol- physicians in real life situation (*n* = 820)	Pain severity, flexibility and quality of life	6 months - improved in all (*p* <.0001), use of other treatments decreased (*p* <.0001)	Not a blind study
				Significant improvements started in 6 weeks
Kertia 2012 [62]	RDB: *C. domestica* 3 × 30 mg (*n* = 34) vs diclofenac 3 × 25 mg (*n* = 39)	COX 2 levels in sinovial fluid at time = 0 and at 4 weeks	All improved no difference between groups	Short trial, no report of change in clinical symptoms
Madhu 2013 [70]	RSBP: NR-INF-02 1 g vs glucosamine 1.5 g vs NR-INF-02 + glucosamine vs placebo	VAS and WOMAC	6 weeks - all treatments showed significant improvement over baseline and placebo	Small study (<30/ group) and over short time period. NR-INF-02 is curcuminoid free extract of *C. longa*
*Avocado-Soybean Unsaponifiables* (piascledine)^b^				
Blotman 1997 [82]	RDBP: ASU (*n* = 83) vs placebo (*n* = 80)	Lequesne’s, Initially all groups received NSAID	6 months - Lequesne’s improved and NSAID use decreased	Over time pain similar in both groups
Maheu et al. 1998 [81]	RDBP: piascledine 300 mg (*n* = 85) vs placebo (*n* = 79) (knee and hip OA)	Lequesne’s, VAS for pain, and NSAID usage	6 months + 2 month follow up. Decrease in Lequesne’s (*p* <0.001), pain (*p* <0.003) and NSAID usage.	Improvement more marked in hip OA.
Lequesne et al. 2002 [95]	RBDP: piascledine 300 mg (*n* = 55) vs placebo (*n* = 53)	JSW, VAS pain, global assessment	2 years - no statistical difference in JSW or clinical parameters.	Posthoc analysis - when OA was severe, ASU slowed the disease progression
Pavelka et al. 2010 [96]	RDB: piascledine 300 mg vs chondroitin 1200 mg (*n* = 263)	WOMAC, Lequesne’s, VAS, global assessment, use of rescue medication	6 months + 2 month follow up. All parameters improved during treatment. Stabilized or improved in the follow up. No differences between groups.	Statistical significance not achieved due to large variability.
Maheu et al. 2014 [97]	RDBP: piascledine 300 mg (*n* = 345)	JSW, WOMAC, Lequesne’s	3 years - fewer progressors in the ASU group. No differences in clinical measurements.	
*Boswellia serrata*				
Kimmatkar et al. 2003 [84]	RDB crossover: *Boswellia serrata* extract vs placebo (*n* = 30)	Knee pain, inflection, walking distance, frequency of swelling, radiology	8 weeks - 3 weeks washout 8 weeks crossover. Significant improvement in all parameters except radiology	Small group size
Sengupta 2008 [85]	RDBP: 5-Loxin (*B. serrata* extract enriched with 30 % AKBA) (*n* = 75)	Pain and function VAS, WOMAC and Lequesne’s	90 days - improvement in stiffness, function and pain scores, decreased MMP-3 (*p* <0.0001).	Serum biochemistry also improved, bioavailability of AKBA is low
Sengupta et al. 2010 [86]	RDBP: Aflapin 100 mg vs 5-Loxin 100 mg vs placebo (*n* = 20/group)	WOMAC, Lequesne’s, VAS, and serum biochemical, hematological and urine changes	90 days - improvement in pain and physical function in both treatment groups.	Small study over short time. Aflapin inhibits MMP-3 and ICAM-1
Gupta et al. 2011 [98]	Open: Shallaki tablet (6 g/d) or tablet and ointment together (*n* = 56 total)	Pain, stiffness and swelling, mental state (Jung scales). Radiology, hematology and biochemistry	2 months - both groups reported significant improvements in pain, stiffness and swelling and by radiology	Not a blind study. No control group.
				Measurements were subjective and results not clear
Vishal et al. 2011 [88]	RDBP: Alfapin vs placebo (*n* = 30/group)	WOMAC, Lequesne’s and VAS for pain and serum biochemical, hematological and urine changes	30 days - significant improvement in pain and function but not in biochemistry	Small short trial to assess safety of new formulation
Kulkarni et al., 1991 [76]	RDBP crossover: Articulin-F capsule (*W. somnifera, B. serrata C. longa* Zinc complex) (*n* = 42)	Pain, stiffness, grip strength, Ritchie articular index, disability score, radiology.	3 months 2 weeks washout crossover 3 months - pain and disability were significantly improved over placebo.	Small prospective study.
Chopra et al. 2004 [99]	RDBP: RA-11 (*W. somnifera, B. serrata, Z. officinale,* and *C. longa)* vs placebo (*n* = 90)	WOMAC, VAS and hematological, urine and biochemical tests	32 week - WOMAC, VAS improved over placebo	Very high dropout rate
Chopra et al. 2013 [89, 100]	RDBP: Glucosamine vs celecoxib vs SGCG^c^ vs SGC (*n* = 440)	Weight bearing pain modified WOMAC	24 weeks - improvement in pain and function in all groups. For WOMAC pain, SGCG worked marginally better than SGC.	Some patients had adverse hepatic effects of SGCG and SGC.

^a^The trials types were random single blind (RSB), random double blind with or without placebo (RDBP, RDB) or open

^b^Studies supported by Laboratoires Expanscience, Courbevoie, France)

^c^SGCG capsule (400 mg) contained *Zingiber officinale, Tinospora cordifolia, Phyllanthus emblica and B. serrata*. The SGC capsule (400 mg) was similar to SGCG (both for content and quantity) except for the absence of *B. serrata* extract and a higher quantity of other ingredients

A major criticism is that most studies did not report curcumin levels in the patients. This is important because curcumin absorption has been reported to be extremely poor when it is used alone [66–69]. To further complicate matters, one study reports that even a curcuminoid free polysaccharide rich extract (NR-INF-02) of *Curcuma longa* may be efficacious against OA (Table 1) [70].

There have been several attempts to improve the bioavailability of curcumin. One study examined the effects of Meriva (curcumin plus phosphatidylcholine for better bioavailability) (Table 1) [71]. Patients (*n* = 100) with mild OA were recruited. They were already under various treatments. The control group continued with only the current treatment but Meriva (2 × 500 mg/day-totalling 200 mg curcumin) was added to the study group. After 8 months, the control group showed an improvement on the WOMAC scale but the treatment group performed much better. The mean treadmill test distances changed from 82.3 to 156 m/6 min in the control group but the increase was much greater in the treatment group (from 77.3 to 344 m/6 min). All the inflammatory markers (IL6, IL1β, sVCAM-1, sCD40L, ESR) were significantly decreased (*p* <0.05) in the study group but not in the control group. Another study reported the effects of Meriva and glucosamine (*n* = 63) vs chondroitin and glucosamine (*n* = 61) in patients with mild OA [66]. The patients improved more with Meriva plus glucosamine than with chondroitin plus glucosamine in WOMAC scores and in the treadmill test. No adverse effects were reported. However, this was not a randomised blind study. It is pointed out that the levels of curcumin in the patients were not reported in either study using Meriva [66, 71]. Another study mixed curcumin with polysorbate for better bioavailability [67]. Curcumin (42 mg/capsule, 2 × 3/day) was given for 3 months. The treatment decreased collagen II but had no significant effects on pain or other biomarkers measured. In another multicenter larger study this combination was also beneficial for pain and flexibility [72]. However, curcumin levels in the patients were not reported in either study. An intravenous infusion of high concentrations of liposomes containing curcumin altered the shape of erythrocytes [73].

One study determined the effect of piperine (inhibits hepatic and intestinal glucuronidation) on the bioavailability of curcumin [69]. After a dose of 2 g curcumin alone, the curcumin levels in the serum were near or below the detection limit. In contrast, a co-administration of 20 mg piperine increased the bioavailability by 20-fold (*p* <0.01). It was concluded that piperine enhanced the serum concentration, extent of absorption and bioavailability of curcumin with no adverse effects. A pilot random double blind study with 53 patients was conducted on the effects of curcumin plus bioperine (3 × (300 mg curcumin + 5 mg piperine)/day) or a placebo for 6 weeks (Table 1) [68]. The treatment led to significant improvements in pain relief and function compared to the placebo. However, there are three concerns with this study. There was no control group without piperine, curcumin levels were not determined, and there was no control group with piperine alone. Therefore, the results are difficult to interpret. An in vitro study on chondrocytes from an OA patient showed that piperine alone can abrogate the IL1-beta-induced overexpression of inflammatory mediators [74].

Curcumin has been reconstituted with non-curcuminoid components of turmeric into a proprietary preparation termed BCM-95CG or Biocurcumax [75]. Biocurcumax increased the oral bioavailability of curcumin when compared to curcumin alone or curcumin plus lecithin. However, there are no reports using this preparation for OA.

Studies on the management of knee OA have also been conducted using *Curcuma* extracts in combination with other substances (Table 1). One trial used the combination of roots of *Withania somnifera*, the stem of *Boswellia serrata* and rhizomes of *Curcuma longa* and a zinc complex. There was a significant improvement in pain relief and function [76]. Ainat - a preparation containing devil’s claw, turmeric and bromelain also showed a clinically relevant improvement in acute and chronic pain [77].

### Avocado-soya extract

Avocado and soybean oils are used for manufacturing soap and the unsaponifiable fraction from these oils is termed avocado/soybean unsaponifiable (ASU). ASU has been tested in the management of OA. ASU contains phytosterols, β-sitosterol, campesterol, and stigmasterol, fat soluble vitamins, triterpene fatty acids and possibly furan fatty acids, but the identity of the active components in it is unknown [78]. Several formulations of ASU are available on the market as supplements. Piascledine, which contains the unsaponifiables as one part from avocado and two from soybean, is a unique patented preparation [79]. In articular chondrocyte cultures ASU may modulate NFκβ levels to inhibit inflammatory cytokines and stimulate collagen synthesis [78]. Animal studies also support its benefits in OA.

A PubMed search in August 2015 with the words “avocado/ASU” and “osteoarthritis” and “knee” showed 25 entries of which five were human clinical trials on pain and function of OA in hip and knee (Table 1) . Literature on the human trials of ASU in hip and knee OA, and for the rationale of this therapy has been reviewed recently [78]. It is pointed out that there may be a conflict of interest in these studies. Also, one should consider that soybean protein alone may be beneficial for OA and this complicates the interpretation of the benefits of ASU [80].

The effect of ASU on patients with hip (*n* = 50) or knee (*n* = 114) OA was examined in a randomised, double blind, placebo controlled, multicenter trial with a 6 month treatment period (Table 1) [81]. After the 6 months its efficacy was greater than that of the placebo (*P* <0.001 for intergroup difference at month 6). The decrease in the Lequesne index (from 9.7 ± 0.3 to 6.8 ± 0.4) was significantly greater in the ASU group than in the placebo group (from 9.4 ± 0.3 to 8.9 ± 0.4). Fewer patients treated with ASU required NSAIDs (48 %) than those in the placebo group (63 %) (*p* = 0.054). The improvements appeared to be more marked in patients with hip than those with knee OA. Another similar study confirmed these findings [82]. In yet another study, the effects of two doses (300 or 600 mg daily) of ASU were compared in patients with knee OA over 3 months (Table 1) [83]. Both doses were effective. At day 90, NSAIDs and analgesics intake decreased to less than half in 71 % patients receiving ASU (300 mg or 600 mg) compared to 36 % in the placebo group (*p* <0.01). The Lequesne’s index dropped by 3.9, 2.9 and 1.9 points with 600 mg ASU, 300 mg ASU and placebo, respectively (*p* <0.01).

### Boswellia

Resins from trees of *Boswellia serrata*, and other species of this genus, have been used for arthritis and other diseases in Ayurvedic medicine since ancient times in India where it is termed shallaki or salai (http://en.wikipedia.org/wiki/Boswellia_serrata). In Europe, it is known as olibanum or Frankincense which is mentioned several times in the Bible (http://www.threemagi.com/frankincense.html). *Boswellia* resins contain several different boswellic acids such as beta-boswellic acid, keto-beta-boswellic acid, and acetyl-keto-beta-boswellic acid (AKBA). AKBA is an inhibitor of the lipoxygenase pathway and is suggested to have anti-inflammatory properties [84].

A PubMed search in August 2015 with the words “Boswellia/shallaki/salai/AKBA” and “osteoarthritis” and “knee” showed 18 entries of which only nine were clinical trials, on *Boswellia* alone or in combination with other substances, with different diagnostic measurements, durations, controls, blindness and conflicts of interests. This included several proprietary preparations from *Boswellia* extracts (Table 1). Most studies examined mainly pain relief or improvements in function. The *Boswellia* extract preparations aflapin and 5-loxin were both shown to be beneficial in arthritis although the studies used small numbers of patients, were conducted over short periods only, and did not meet all rigorous criteria [85–88]. Nevertheless, almost all the studies have shown some benefit for OA (Table 1). In some studies, preparations combinations of *Boswellia* and several other substances were used (Table 1). A small random double blind crossover study conducted in 1991 found that articulin (combination of *W. somnifera* 450 mg, *B. serrata* 100 mg, *C. longa* 50 mg, Zinc complex 50 mg) improved pain and functional symptoms of OA (Table 1) [76]. However, a literature search showed that the study was not repeated with a larger size. A more extensive study compared two drugs termed SGCG and SGC, both containing several herbs but only SGCG containing *Boswellia* (Table 1) [89]. Both drugs were effective against OA symptoms but the drug containing *Boswellia* was marginally more effective in reducing the WOMAC pain symptoms.

### Ayurvedic and Chinese medicines

Several herbal products containing antioxidants are used in Ayurvedic medicine. For example, roots of *Withania somniferum* are used to prepare ashwagandha. It contains withanaloids, most importantly withaferin A [90]. *Tinospora cordifolia* (Guduchi) is considered a divine herb and it contains diterpenoids termed tinosporides [91]. *Emblica officinalis* (or *Phyllanthus emblica*) is termed amla or amlaki and is rich in vitamin C and emblicanins A and B [92]. *Zingiber officinale* (Ginger) root is not only used extensively in cooking but also has several medicinal antioxidants. The analysis of volatile oils of fresh and dried ginger showed camphene, *p*-cineole, α-terpineol, zingiberene and pentadecanoic acid as major components [93]. There are several studies demonstrating the usefulness of these antioxidants in OA. A major complication in comparing their usefulness comes from a lack of an exact parallelism between the pathological classifications in Ayurvedic system versus the Western system of medicine. Further, several studies have used only small populations, have fewer controls and their methods of analysis may lend to bias. The characteristics of the studies are presented in a recent review along with their risk of bias according to Cochrane risk of bias tool [38]. Their study included 19 randomized and 14 non-randomized controlled trials on 12 different drugs and three non-pharmaceutical interventions. They concluded that “The drugs Rumalaya and Shunti-Guduchi seem to be safe and effective drugs for treatment of OA-patients, based on these data. However, several limitations relate to clinical research on Ayurveda. Well-planned, well-conducted and well-published trials are warranted to improve the evidence for Ayurvedic interventions.” However, part of the problem is that these are very complex proprietary preparations with undisclosed exact compositions. For example, Rumalaya contains Mahayograj guggul, Shankha bhasama, purified Shilajeet, Swarnamakshik bhasama, Latakasthuri and Shallaki, Jaya galangal, Licorice and Tinospora Gulancha. Chemical compositions of each of these is not exactly known.

Chinese herbal medicines have been used for treating OA in China for centuries. The most commonly prescribed OA medicines in Taiwan are Du-huo-ji-sheng-tang plus Shen-tong-zhu-yu-tang, and the triple-drug combination was Du-huo-ji-sheng-tang, Gu-sui-pu (Drynaria fortune (Kunze) J. Sm.), and Xu-Duan (Himalaya teasel) [37]. These are all polyplant preparations which have not been critically evaluated as per today’s criteria. One study examined the preparation Duhuo Jisheng Tang for treating OA of the knee [94]. This preparation contains parts from 15 different herbs and its chemical constituents include coumarins, phytosterols, polysaccharides, flavanoids, monoterpene glycoside, galloyl glucoses, saponins, ginsenosides and alkaloids. Patients (*n* =68) of both genders and 59.2 ± 10.2 years of age were given a dose 2.5 g twice daily for 4 weeks. There was a highly significant improvement in the WOMAC scores (pain, stiffness and function) and in visual analog scale for pain. As noted, the study used a small number of subjects for a short duration and there was no placebo group.

### Synopsis

OA is a disease of inflammation of large articulate joints with the tibiofemoral joint inflammation being more prevalent. There is no known cure for this disease but considerable progress has been made in joint replacement surgery. The pharmacological strategies for coping with OA include glucosamine plus chondroitin sulfate, hyaluronic acid or corticosteroid injections and NSAIDs.

The pathophysiology of OA is consistent with the hypothesis that the inflammatory molecules and ROS increase with the onset of the disease. Therefore, it is logical to think that antioxidant supplements may be of some benefit. Curcumin which is isolated from *Curcuma longa* may be efficacious for pain relief and function retention in OA patients. Several products which increase oral bioavailability of curcumin may be promising. Another beneficial product may be ASU. Boswellia resins may also be beneficial and proprietary preparations derived from it and containing AKBA may also have good efficacy. All of these products have fewer adverse effects than the chronic use of NSAIDs. Ayurvedic and ancient Chinese medicines may also be beneficial.

### Critical appraisal

The problem of defining OA has been elegantly discussed in a Clinical Viewpoint [2]. Common understanding today is that OA is a sequential process: loss of proteoglycans on the cartilage surface, death of chondrocytes in deeper layers, hypertrophy and aggregation of remaining chondrocytes, formation of surface cracks parallel to articular face, flow of synovial fluids into these defects, immune reactions due to cartilage fractures, penetration of synovial fluid into defects and cyst formation, osteophyte formation leading to joint crepitus and stiffness, restriction of movements and narrowing of intra-articular space. Pain severity increases with the various stages. One of the problems is that radiographic imaging may show joint space narrowing without any loss in range in motion or any other symptoms. Radiographic imaging does not show cartilage damage and hence MRI, ultrasound, and optical coherence tomography have been introduced recently for diagnosis. However, most studies in drug trials restrict to methods such as WOMAC, VAS or Lequesne index using questionnaires or walking or stair climbing. Each of these indices contains a large number of items which deter from a focus on the definition of the disease. With the exception of quantitative measures such as distance walked in 6 min, the methods are highly subjective and a source of bias due to their subjectivity. Stair climbing is a good functional measure but it may often depend on cardiorespiratory abilities in addition to osteoarthritis.

A major hurdle in today’s evidence based medicine is money. When money is to be made such as in proprietary or patented drugs, a large investment is expected in the trials. However, when a traditional supplement is to be tested, financial investments may be small and hence large trials do not occur. Conflicts of interest - declared or undeclared may also come about due to such investments. For example, turmeric has been used in Ayurvedic and Chinese medicine for arthritis since ancient times. Curcumin was isolated from it in 1815 [61]. Curcumin has been tested extensively for its benefits in arthritis and other diseases. It is interesting that the bioavailability of this compound is extremely low when given orally in powdered forms in capsules. Yet, when consumed in large doses, it is beneficial for OA. Commercial proprietary preparations using curcumin have been developed to increase its bioavailability. It is ironic that one of the products is made from reconstituting curcumin with curcumin free components from turmeric. Another interesting product contains curcumin and piperine which can be isolated from black pepper. It enhances the bioavailability of curcumin. These products are being tested for their efficacies. The Indian housewife uses turmeric and black pepper routinely in cooking. Yet, the efficacy of this product will not be tested since there is nothing proprietary in it. Even if money were available, taste and colour of turmeric and black pepper would deter a randomized double blind clinical study. Placebos would also be hard to find. This example reflects the type of difficulty one has in evaluating the usefulness of the literature in this area. These difficulties often muddle the clarity and usefulness of the conclusions.

## Recommendations

This review shows that there is some evidence for benefits of antioxidant supplements in pain relief and function in knee OA. These supplements with the most evidence include curcumin, avocado-soya bean unsaponifiables, *Boswellia* and several preparations used in Ayurvedic and Chinese medicine. These should be tested further and used, at least, to decrease the use of NSAIDs which have more adverse effects.

Ancient medicine should be tested as such rather than proprietary products made from them. It should be assessed whether diet with turmeric and black pepper results in sufficient levels of serum curcumin. Promotion of dietary habits may be more economical and of longer term benefit than the development of products made from extracts used in ancient medicine.

## Abbreviations

AKBA: acetyl-keto-beta-boswellic acid
ASU: avocado-soya bean unsaponifiables
IL: interleukin
NFκβ: nuclear factor kappa-beta
NSAIDS: nonsteroidal anti-inflammatory drugs
OA: osteoarthritis
ROS: reactive oxygen species
SOD: superoxide dismutase
TNF-α: tumour necrosis factor alpha
VAS: visual analog scale
WOMAC: Western Ontario McMaster Index
